# Health Tract No. 348

**Published:** 1868-11

**Authors:** 


### NIGHT AIR.

There can be no pure air in any inhabited house, even if it be without a roof, and any out-door air, with some exceptions perhaps, is purer, softer and better, in the same locality, other things being equal. The exceptions referred to are in marshy localities in warm weather. But the reader must guard himself against fanatical errors, or he will lose his life. The reapers of the marshes about Rome used to come into the city at night and sleep in door-ways or on the steps of public buildings, because they knew very well that if they slept in the fields they would sicken and die. Fifty years ago, it was certain death for a stranger or countryman to sleep in the city of Charleston a single night in summer; and yet they could sleep there in the day-time with impunity. Thousands have become fatally diseased by sleeping at Aspinwall or Panama, a single night, yet to remain there in the day-time is not dangerous. Thousands who live on the banks of rivers know that in the autumn those who sleep with the windows hoisted during the night will become sick in a few days; hence it depends on circumstances whether it is wise to sleep at night with the windows hoisted; and, as in traveling, we may not be acquainted with the circumstances which surround us, the safer plan is, to be wary of the night air and keep the outer window closed and let the door of the sleeping apartment be left open, or the fire-place be unobstructed; or, let a lamp or a little fire be burning in it to create a draft up the chimney; and considering the danger of sudden changes or of cold damp winds arising, it is rather better to accustom ourselves to have the outer windows of our chamber closed when we go to bed; but if there is no fire-place or the door cannot be left open, then by all means hoist the window, and if the draft comes in upon you, stretch a garment within two or three inches of the opening, which will direct a part of the draft towards the ceiling, and part towards the floor.

Franklin was an enthusiastic advocate of breathing fresh air; he luxuriated in hoisted windows, and by sitting at a hoisted window he contracted a malady which killed him, according to Thomas Jefferson, such is the tendency of the human mind to run to extremes, to become idiotic on hobbies; circumstances modify every thing practical; facts are often mischievous falsehoods from not taking into view all the parts.

The Ferroe Islanders live longer than any other people on an average, and yet they are the filthiest people in the world about their habitations; the girls of Brittany have the longest, glossiest and most luxuriant hair that can be found, and yet they are dirty in their persons, and pay less attention to combing and ventilating their hair than others; in fact they keep it tied out of sight with a handkerchief for weeks together, a tangled mess; these are partial facts; the Ferroe Islanders are out on the sea all summer and are only at home in their petty houses in the coldest months in the year, when there is no risk from the poison and heat; and the girls of Brittany do not lard, and pull, and strain and frizzle each particular hair out of its health and life and root, as we do.

